# Mitigation of Ischemia-Reperfusion Injury and Improvement in Overall Graft Viability by Hypothermic Pulsatile Perfusion with Molecular Hydrogen Is Associated with Trx-1/HO-1 Activation in a Non-Survival Ex Vivo Swine Model of Donation-After-Circulatory-Death Kidney Preservation and Transplantation

**DOI:** 10.3390/ijms27114931

**Published:** 2026-05-29

**Authors:** George J. Dugbartey, Cora England, Tamara S. Ortas, Mahmoud Richard-Mohamed, Larry Jiang, Talal Shamma, Martin Igbokwe, Ali Bozaci, Juan Gonzalez Oyarzun, David Seok, Saeeda A. Zainul, Lori Harrow, Monica Freeman, Renee Lindo-Anu, Aushanth Ruthirakanthan, Abdullah Alfaifi, John Wang, Patrick McLeod, Aaron Haig, Christopher Bonham, Alp Sener

**Affiliations:** 1 Matthew Mailing Center for Translational Transplant Studies, London Health Sciences Center, Western University, London, ON N6A 5A5, Canada; gdugbart@uwo.ca (G.J.D.);; 2 Department of Surgery, Division of Urology, London Health Sciences Center, Western University, London, ON N6A 5A5, Canadadavid.seok@lhsc.on.ca (D.S.);; 3 Multi-Organ Transplant Program, London Health Sciences Center, Western University, London, ON N6A 5A5, Canada; 4 Department of Physiology & Pharmacology, Western University, London, ON N6A 5C1, Canada; jwan2588@uwo.ca; 5 Department of Physiology & Pharmacology, Accra College of Medicine, Accra P.O. Box CT9828, Ghana; 6 Department of Microbiology & Immunology, Western University, London, ON N6A 5C1, Canada; 7 Department of Transplant Research and Innovation, King Faisal Specialist Hospital and Research Centre, Riyadh 11211, Saudi Arabia; 8 Department of Pathology, Schulich School of Medicine & Dentistry, University of Western, London, ON N6A 5C1, Canada; 9 Department of Biochemistry, Siebens-Drake Research Institute, Western University, London, ON N6A 5C1, Canada

**Keywords:** kidney transplantation, ischemia-reperfusion injury (IRI), donation-after-circulatory-death (DCD), molecular hydrogen (H_2_), hypothermic pulsatile perfusion

## Abstract

Despite their reduced viability, kidneys from donors-after-circulatory-death (DCD) increase the pool of transplantable kidneys. Molecular hydrogen (H_2_) is emerging as a gas with therapeutic potential against graft injury. We investigated the effect of H_2_ in an ex vivo porcine model of DCD kidney transplantation. Renal arteries of male Yorkshire pigs (*n* = 6) were clamped in situ for 60 min to induce ischemia, and ureters and arteries were cannulated to mimic DCD kidney injury. Upon nephrectomy, kidneys were flushed with UW solution or H_2_-saturated UW solution and then preserved by machine perfusion at 4 °C for 4 h followed by a 4-h reperfusion period with warm autologous blood. Urine and arterial blood samples were collected hourly. H_2_ preserved renal architecture, evidenced by significantly reduced tubular necrosis and renal expression of damage markers, which corresponded with the downregulated renal expression of pro-inflammatory genes compared to the UW-only group (*p* < 0.05). H_2_ also markedly reduced levels of serum creatinine, BUN and intrarenal resistance, while flow rate, creatinine clearance and urine output were significantly higher, which positively correlated with Trx-1 and HO-1 expression in comparison with UW only group (*p* < 0.05). Improvement in renal graft quality and function is associated with Trx-1/HO-1 activation, suggesting preliminary clinical trials in kidney transplantation.

## 1. Introduction

Kidney transplantation has become a routine and effective renal replacement therapy for patients with end-stage renal disease. It presents several advantages over dialysis therapy, such as improved quality of life of patients at a relatively cheaper cost [1]. However, the global shortage of living donor kidneys and the ever-growing list of patients on the transplant waiting list as well as the increasing death toll on the transplant waiting list have necessitated the use of kidneys from deceased donors, including donation-after-circulatory-death (DCD) kidneys [2,3]. Unfortunately, DCD kidneys are associated with poor graft quality and function, as a lack of perfusion due to prolonged warm ischemia increases ischemic injury that is further exacerbated during reperfusion, termed ischemia-reperfusion injury (IRI) [4,5]. IRI is an unavoidable pathological condition that increases the incidence of post-transplant complications such as slow or delayed graft function (DGF) and primary non-function (PNF), leading to acute graft rejection and early graft loss [5,6,7]. Nonetheless, the use of kidneys from deceased donors still represents an important countermeasure to expand the pool of donor kidneys to help solve the global donor kidney shortage crisis.

The expansion of the pool of donor kidneys with DCD kidneys suggests the urgent need to consider the improvement of the transplant protocol, including renal graft preservation strategies to enable the utilization of thousands of kidneys from deceased donors that are discarded annually due to their increased likelihood of post-transplant complications. To address this global concern, molecular hydrogen (H_2_), also known as dihydrogen or hydrogen gas, applied at very low non-combustible concentrations is emerging as a novel therapeutic additive to improve graft preservation technique and transplant outcomes by combating IRI. H_2_ is a tasteless, odorless, non-toxic gas that is widely distributed in nature and participates in many physiological processes [8,9]. Various disease models and treatment studies have shown that H_2_ exerts selective antioxidant function with anti-inflammatory and anti-apoptotic properties [10,11,12,13]. Recent animal models of non-renal transplantation also showed that H_2_ mitigates IRI through its cytoprotective properties, leading to improved graft quality and function and prolonged transplant recipient survival [14,15,16,17]. While there is growing evidence of the therapeutic impact of H_2_ on non-renal grafts, there are relatively few studies on its effect in kidney transplantation. Moreover, the underlying protective mechanisms of H_2_ in these transplant models are poorly understood. Mechanistically, thioredoxin-1 (Trx-1) and heme oxygenase-1 (HO-1) are emerging as important proteins that exhibit cytoprotective effects under stressful conditions by reducing oxidative stress and inhibiting inflammation. However, this pathway has not been studied in kidney transplantation settings. Using a non-survival ex vivo porcine model of DCD kidney transplantation, the present study investigated whether H_2_ is also beneficial in this model and explored the Trx-1/HO-1 pathway as a possible molecular mechanism of protection.

## 2. Results

### 2.1. H_2_ Improved Renal Graft Function During Hypothermic Pulsatile Perfusion and Normothermic Reperfusion

**Figure 1:**
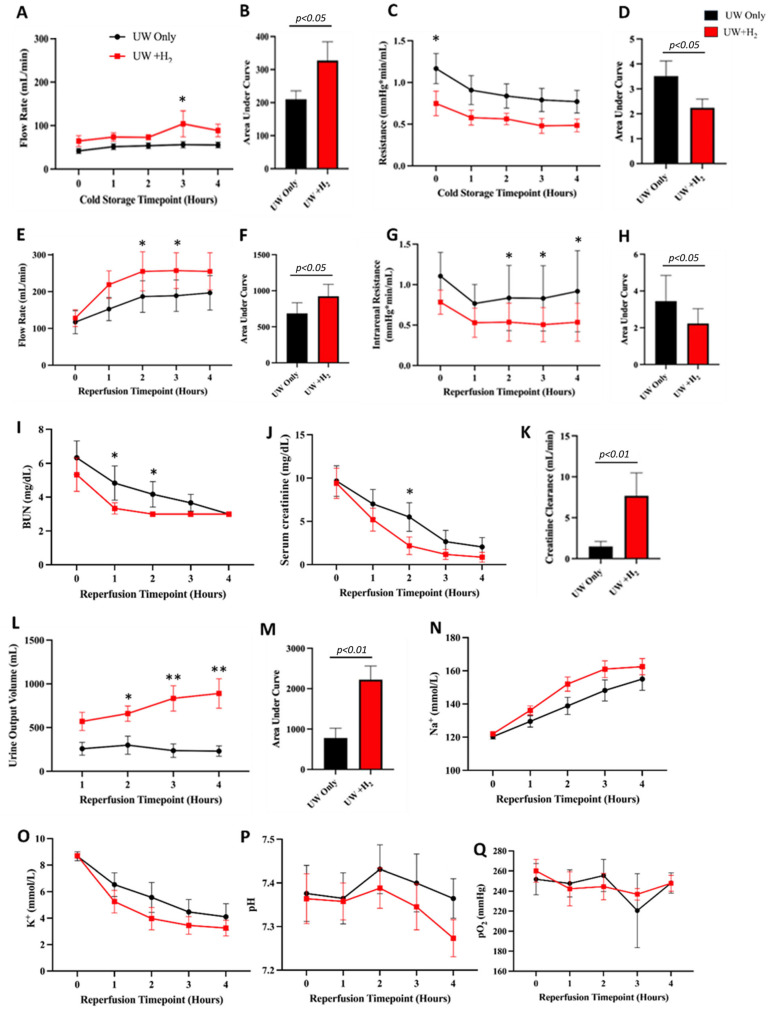
H_2_-saturated UW solution improved renal graft function during hypothermic pulsatile perfusion and reperfusion. (**A**,**B**) Flow rate during hypothermic preservation and its area under curve, (**C**,**D**) Intrarenal resistance during cold storage and its area under curve, (**E**,**F**) Flow rate during reperfusion and its area under curve, (**G**,**H**) Intrarenal resistance during reperfusion and its area under curve, (**I**) BUN, (**J**) Serum creatinine, (**K**) Creatinine clearance, (**L**,**M**) Urine output and its area under curve, (**N**) Plasma Na^+^, (**O**) Plasma K^+^, (**P**) Blood pH, and (**Q**) Arterial oxygen tension (pO_2_). Values are mean ± SEM. */** *p* < 0.05/0.01.

Parameters of renal graft function were measured to evaluate the effect of H_2_ during hypothermic pulsatile perfusion and normothermic reperfusion. Compared to control grafts, renal grafts stored in the H_2_-saturated UW solution showed significantly increased flow rates during cold storage and reperfusion along with corresponding reductions in intrarenal resistance (Figure 1A–H; *p* < 0.05). In addition, blood urea nitrogen (BUN) and serum creatinine in control renal grafts were higher than in H_2_-treated grafts throughout the reperfusion period, with significantly higher values at the end of the first and second hours of reperfusion (Figure 1I,J; *p* < 0.05). This observation corresponded with markedly increased creatinine clearance in H_2_-treated grafts relative to control grafts (Figure 1K; *p* < 0.01). Similarly, H_2_-treated renal grafts produced higher urine volume throughout the reperfusion period compared to control grafts, with significantly higher volumes at the end of the second, third and fourth hours of reperfusion (Figure 1M; *p* < 0.01). Interestingly, both groups of kidneys showed no statistical difference in blood pH, sodium (Na^+^) and potassium (K^+^) levels or arterial oxygen tension (pO_2_) (Figure 1N–Q; *p* > 0.05). Altogether, H_2_ improved renal graft function, as characterized by significantly increased blood flow rate, creatinine clearance and urine output and markedly reduced intrarenal resistance, serum creatinine and BUN.

### 2.2. H_2_ Preserved Renal Graft Architecture Following Hypothermic Pulsatile Perfusion and Normothermic Reperfusion

**Figure 2:**
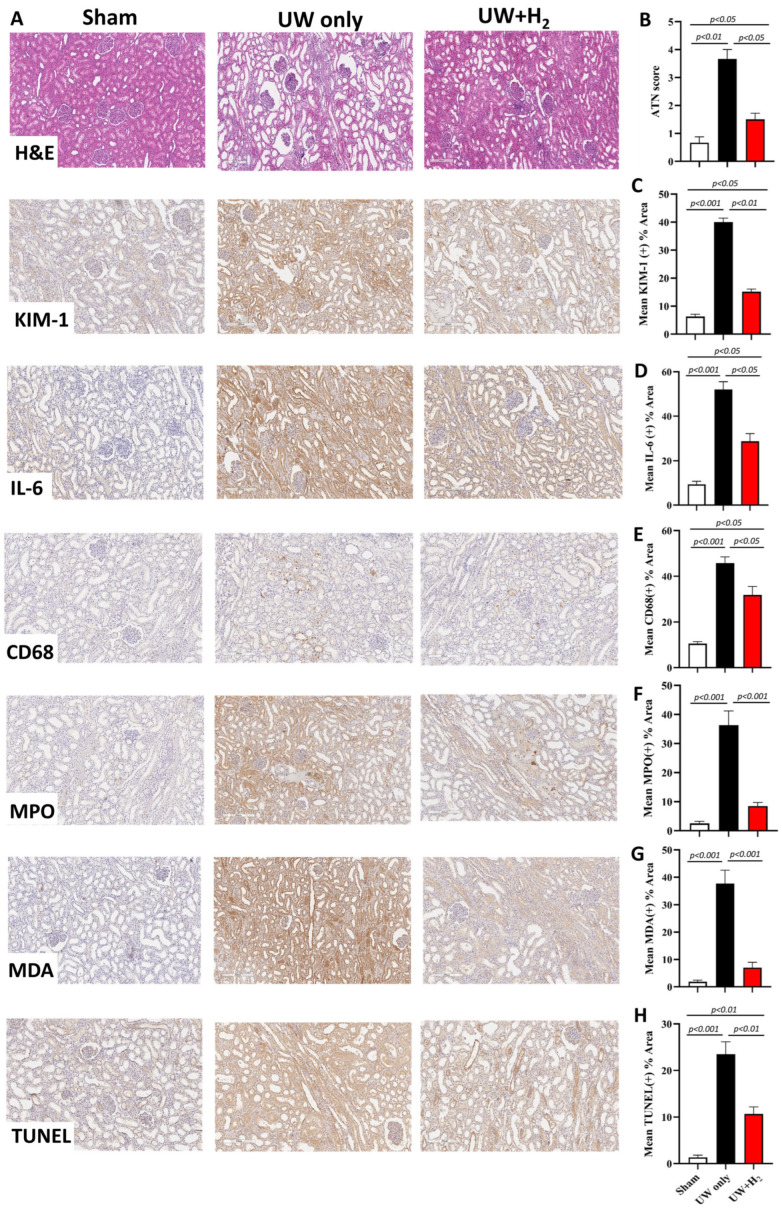
H_2_-rich UW solution preserved renal graft structure. (**A**) Representative images of sham, control and H_2_-treated kidneys showing H&E, KIM-1, IL-6, CD68, MPO, MDA and TUNEL at 100× magnification. Quantitative analysis of (**B**) acute tubular necrosis, (**C**) KIM-1, (**D**) IL-6, (**E**) CD68, (**F**) MPO, (**G**) MDA, and (**H**) TUNEL. Values are mean ± SEM.

**Figure 3:**
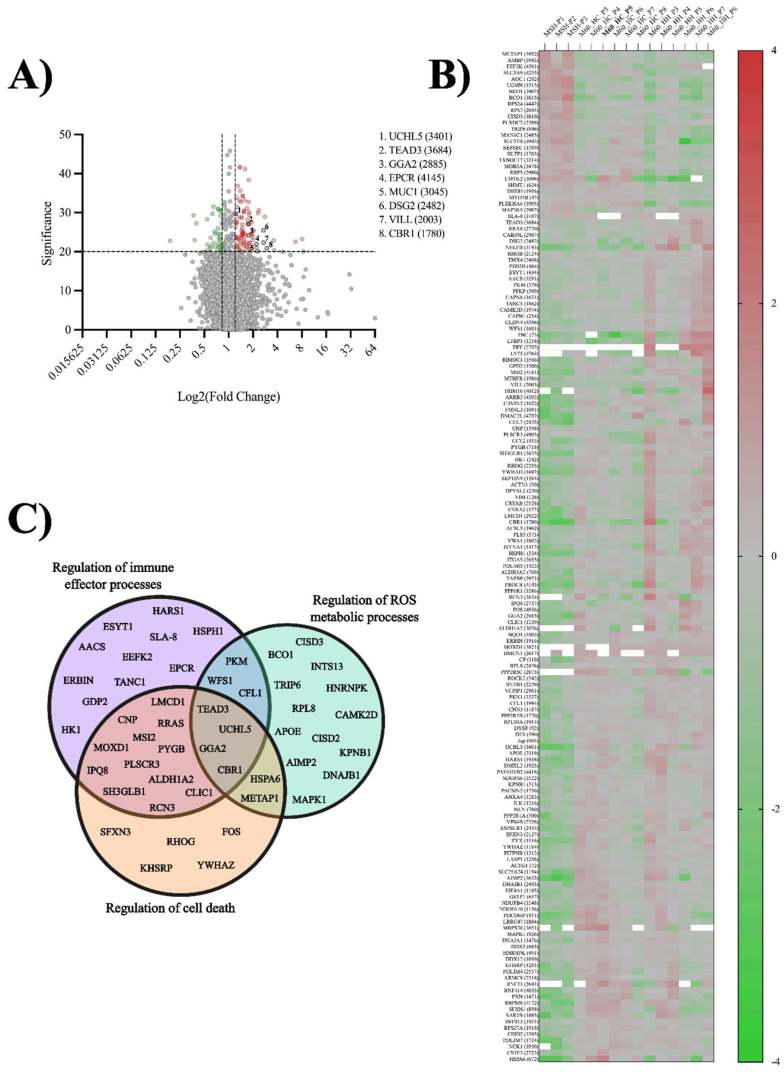
Proteomic analysis reveals upregulated expression of immune regulatory and protective proteins against cellular stress in H_2_-treated renal grafts. Upregulated proteins represented in red and downregulated proteins represented in green. (**A**) Volcano plot displays differentially expressed proteins in H_2_-treated renal grafts compared to control kidneys. Dotted line along *y*-axis represents significance equivalent to *p* < 0.01. (**B**) Heat map shows Z score for each differentially expressed protein in each biological replicate. (**C**) Venn diagram highlights contextually relevant proteins with a significance of *p* < 0.01. Circle color indicative of representative pathway(s), including regulation of immune effector processes, ROS metabolic processes, and regulation of cell death. MSH = sham, M60_HC = control; M60_HH = H_2_-treated.

Histological and immunohistochemical staining were performed to assess the degree of IRI and the impact of H_2_ on renal graft injury. Hypothermic storage and the reperfusion of renal grafts resulted in severe damage in the glomerular and tubular compartments, while H_2_ preserved these renal compartments to a level that is comparable to the sham group (Figure 2A). Quantitatively, acute tubular necrosis scores were significantly higher in control renal grafts compared to sham-operated kidneys (Figure 2B; *p* < 0.01). However, H_2_-treated grafts displayed significantly less acute tubular necrosis relative to control grafts (Figure 2B; *p* < 0.05) and almost comparable levels to the sham-operated group (Figure 2B). Similarly, the expression of KIM-1 (kidney injury molecule) was markedly downregulated in sham- and H_2_-treated kidneys in comparison with control kidneys (Figure 3A,C). The renal expression of other damage markers such as IL-6 (pro-inflammatory cytokine), CD68 (macrophage infiltration), MPO (neutrophil influx), MDA (ROS indicator) and TUNEL (apoptosis) followed the same trend (Figure 2A,D–H). In summary, H_2_ attenuated renal graft injury and preserved graft architecture after hypothermic pulsatile perfusion and normothermic reperfusion.

### 2.3. H_2_ Upregulated Expression of Proteins Conferring Protection Against Renal Graft Injury

To further characterize the impact of H_2_ preservation on renal grafts, we performed proteomic analyses of H_2_-treated grafts and control grafts using LC-MS. A total of 6916 proteins were identified across all samples (H_2_, control, and sham), and those with a significance score ≥ 20 (*p* < 0.01) were considered significant. Of these, 104 proteins were found to be upregulated (fold change ≥ 1.2), represented in red, and 35 proteins were found to be downregulated (fold change ≤ 8.3), represented in green (Figure 3A,B). Broadly, we found the upregulation of 31 more proteins associated with regulating immune effector processes in H_2_-treated kidneys compared to control kidneys (Figure 3C). Protective proteins such as carbonyl reductase 1 (CBR1) and endothelial cell protein C receptor (EPCR), which protect against oxidative stress, inflammation and coagulation, had two- and four-fold greater changes expressed in H_2_-treated kidneys compared to control kidneys. Also, desmoglein-2 (DSG2), mucin 1 (MUC1) and villin-like (VILL), protective proteins against renal cell injury, were upregulated in H_2_-treated tissue and preserved renal graft function better compared to control kidneys. In addition, several proteins responsible for regulating ROS metabolic processes and cell death were also upregulated (Figure 3C). Taken altogether, H_2_ significantly upregulated numerous proteins associated with the preservation of renal graft function and structural integrity.

### 2.4. H_2_ Conferred Renal Graft Protection by Activating Renal Trx-1/HO-1 Signaling Pathway

**Figure 4:**
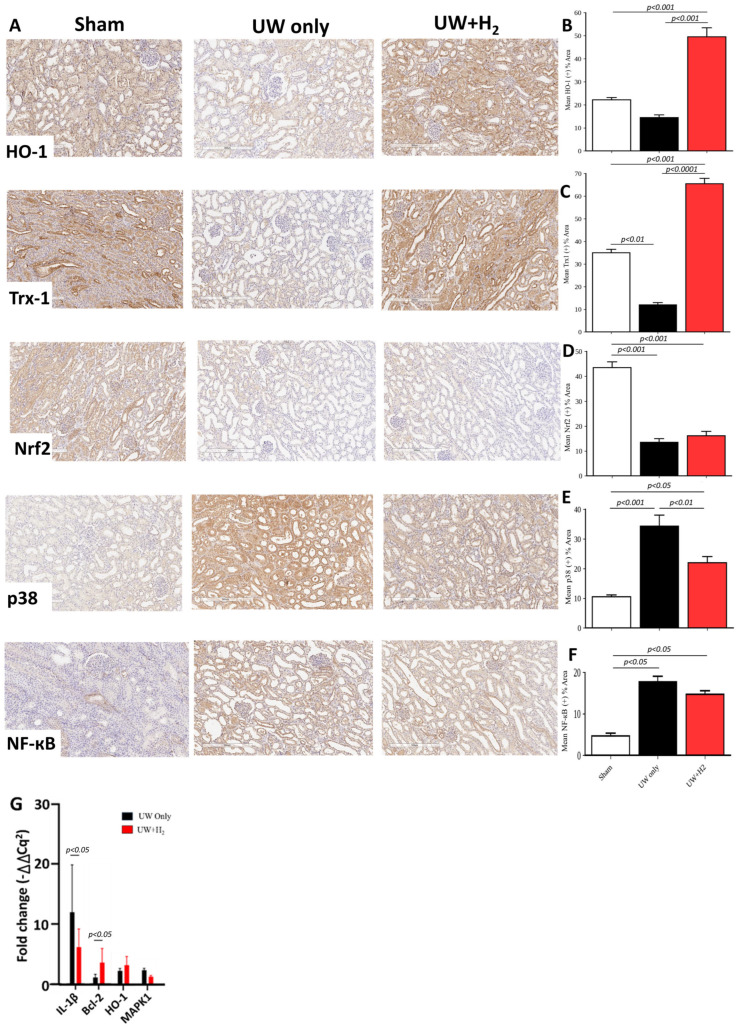
Mechanism of renal graft protection by H_2_. (**A**) Representative images of sham, control and H_2_-treated kidneys showing HO-1, Trx-1, Nrf2, p38, and NF-κB at 100× magnification. Quantitative analysis of (**B**) HO-1, (**C**) Trx-1, (**D**) Nrf2, (**E**) p38, and (**F**) NF-κB. (**G**) qPCR analysis of renal graft homogenates for expression levels of IL-1β, Bcl-2, HO-1, and MAKP1. Genes were normalized against GAPDH. Values are mean ± SEM.

Additional immunohistochemical staining and rt-qPCR were performed to examine the mechanisms by which H_2_ confers renal graft protection during hypothermic storage and normothermic reperfusion. As shown in Figure 4, H_2_ strongly upregulated the renal expression of heme oxygenase-1 (HO-1) and thioredoxin-1 (Trx-1) relative to control grafts (Figure 4A–C; *p* < 0.001). Remarkably, the expression of HO-1 and Trx-1 in H_2_-treated renal grafts were further upregulated significantly compared to sham kidneys (Figure 4A–C; *p* < 0.001). Interestingly, H_2_ had no effect on Nrf2 expression (Figure 4A,D). Additionally, H_2_ also markedly downregulated the renal expression of p38 (Figure 4A,E; *p* < 0.01), with a slight decrease in NF-κB expression compared to the expressions in control grafts (Figure 4A,F). The mRNA expression of these proteins followed a similar trend (Figure 4G). Collectively, H_2_ activated the renal Trx-1/HO-1 pathway, leading to renal graft protection after hypothermic preservation and normothermic reperfusion.

## 3. Discussion

Using a clinically relevant ex vivo porcine model of DCD kidney preservation by hypothermic pulsatile perfusion and transplantation, the present study investigated the impact of H_2_ and its underlying mechanisms of action. Notably, our result showed that the flushing and preservation of renal grafts with H_2_-saturated UW solution for 4 h followed by normothermic reperfusion for another 4 h mitigated IRI and improved the overall viability of renal grafts. This was characterized by increased flow rates during hypothermic pulsatile perfusion as well as increases in blood flow rate, creatinine clearance and urine output during normothermic reperfusion, with reduced intrarenal resistance, serum creatinine and BUN. In addition, H_2_ made the renal grafts rinsed faster following graft procurement, and preserved renal graft architecture as evidenced by reduced interstitial congestion and hemorrhage, lower acute tubular necrosis scores, reduced renal expression of KIM-1, decreased influx of macrophages and neutrophils, cytokine production as well as reactive oxygen species (ROS) generation and apoptosis, all of which were comparable to kidneys from the sham group. Although perfusion pressure was controlled in the present study, the differences in flow rate and urine output in control and H_2_-treated kidneys suggest impaired autoregulatory capacity in injured DCD kidneys, and that H_2_-treated kidneys may not simply be hyperperfused; rather, H_2_ preserves vascular responsiveness and reduces ischemia-induced vasoconstriction and endothelial dysfunction. Thus, our observation suggests that H_2_ preserves vascular resistance, endothelial function, and tubular integrity rather than pressure-driven perfusion alone.

Our finding is consistent with a recent miniature pig model of DCD kidney transplantation in which storage of renal grafts in H_2_-containing extracellular-type trehalose-containing Kyoto (ETK) solution at 4 °C for 4 h prevented primary nonfunction by restoring renal blood flow (RBF) and urine production at sacrifice on post-operative day 6 (POD6) compared to H_2_-free control grafts without the detection of RBF and urine production [18]. This is also similar to another report in a rat model of chronic allograft nephropathy in which H_2_ increased creatinine clearance and reduced proteinuria [19]. The improvement and restoration of RBF and urinary excretory function by H_2_ in the present study and in the miniature pig model, respectively, as well as the increased creatinine clearance, suggest that H_2_ possesses a vasodilation property, as it activated mitochondrial ATP-sensitive potassium (K_ATP_) channels (a type of potassium channel whose activation and opening promotes vasodilation and regulates vascular tone) and mediated cardioprotection in a canine model of IRI [20]. This explanation supports recent experimental and clinical findings showing that H_2_ reduces endothelial alterations, restores vascular function and enhances vascular health in various disease conditions [21,22,23,24] by inhibiting oxidative stress and inflammation and activating beneficial cellular signaling pathways such as NO/cGMP, PI3K/Akt, and AMPK/mTOR pathways [24,25,26,27,28]. Although we did not investigate these signaling pathways, it is possible that the mitigation of IRI in the present study was partly via the activation of these signaling mechanisms. Also, our observation of faster rinsing by the H_2_-rich UW solution may be explained by the result from the study by Abe et al. [29], who reported the dilation of renal peritubular capillaries by H_2_ during rinsing with a H_2_-rich UW solution. This further supports the reports that H_2_ possesses a vasodilation property and enhances vascular health.

In addition to improving renal graft function, the findings from our work also show that the preservation of normal tissue structure and the overall renal graft protection by H_2_ against IRI is partly due to its selective antioxidant, anti-inflammatory and anti-apoptotic properties, as observed in the downregulated expression of MDA (indicator of ROS production and oxidative stress), interleukin-6 (IL-6; pro-inflammatory cytokine), CD68 (macrophage marker), myeloperoxidase (MPO; neutrophil marker), nuclear factor kappaB (NF-κB; a transcription factor), p38 (a member of mitogen-activated protein kinase [MAPK] family) and TUNEL (apoptosis) in our immunohistochemical staining, which positively correlated with mRNA expression levels of IL-1β (another pro-inflammatory cytokine) and MAPK1 (also called ERK2; a member of MAPK family), and the upregulated expression of the anti-apoptotic gene, Bcl-2. This result is in agreement with reports from a previous kidney transplant model showing attenuation of tubular injury, tubular apoptosis, macrophage infiltration and interstitial fibrosis in renal grafts preserved in a H_2_-saturated UW solution at 5 °C for 24 h, thereby reducing oxidative stress and inflammation, and thus contributing to improved renal graft function and survival in a rat model of syngeneic kidney transplantation [29], as well as in various rat models of renal IRI [30,31,32]. Interestingly, while the syngeneic kidney transplant model used a longer preservation period (24 h), we chose a much shorter preservation time (4 h; shorter than preservation time used clinically) in order to establish a proof of principle for treatment to ensure that animal models of H_2_ therapy worked prior to moving on to longer preservation times. It is important to note that NF-κB and MAPK are principal mediators of inflammation in various human diseases. While stressful stimuli such as cold ischemia and reperfusion activate the nuclear translocation of NF-κB to induce the expression of pro-inflammatory cytokines such as IL-1β and IL-6—which represents one of the first inflammatory responses to hyperoxia during reperfusion, leading to neutrophil activation and infiltration, and oxidative damage, as observed in the present study—members of the MAPK family (e.g., p38 and MAPK1), on the other hand, transmit extracellular signals that activate the production of these pro-inflammatory cytokines [33,34]. Therefore, our finding suggests that H_2_ offers a possible way to attenuate transplantation-induced inflammation and its downstream cascade while simultaneously activating antioxidant and anti-apoptotic pathways, as the anti-apoptotic effect of Bcl-2 is known to be via antioxidant activity with reduced ROS production [35]. By downregulating the expression of pro-inflammatory genes and reducing ROS generation while upregulating anti-apoptotic genes in the renal grafts, our finding further suggests an intimate crosstalk between inflammation, oxidative stress and apoptosis, which are modulated by H_2_ to preserve graft integrity under the stressful conditions of cold ischemia and reperfusion in organ transplantation. Moreover, our proteomics data showed a significant upregulation of endothelial cell protein C receptor (EPCR) and carbonyl reductase 1 (CBR1) proteins in H_2_-treated kidneys, which partly contributed to preservation of renal structural and functional integrity. It is worth noting that, while EPCR has been previously reported to preserve renal function by downregulating the expression of neutrophils, reducing the production of pro-inflammatory cytokines such as interferon-gamma (INF-γ) and interleukin-1beta (IL-1β) as well as the levels of circulating pro-inflammatory chemokines (CXCL1 and CXCL2) in rat models of sepsis [36,37], the induction of CBR1 by Nrf2, in turn, activated antioxidant pathways, leading to reduced ROS production and oxidative stress in a mouse model of hepatic IRI and liver biopsies in clinical liver transplantation [38]. Furthermore, our proteomic analysis revealed a substantial upregulation of protective proteins such as desmoglein-2 (DSG2), mucin 1 (MUC1) and villin-like (VILL) against renal cell injury, as well as other protective proteins (e.g., TEAD3, UCHL5, GGA2), which are essential for cell survival and the preservation of antioxidant status in H_2_-treated kidneys, thus culminating in the preservation of renal graft structure and function compared to control kidneys.

There is burgeoning evidence suggesting modulation of various cellular signaling pathways underlying the protective effect of H_2_, which are interrelated with its antioxidant, anti-inflammatory and anti-apoptotic properties. Along these signaling mechanisms, an important novelty in the present study is the finding that the observed renal graft protection by H_2_ is associated with the activation of the Trx-1/HO-1 pathway. Thioredoxin-1 (Trx-1) is an important intracellular redox-active protein whose activation and nuclear translocation from the cytosol is in response to oxidative stress conditions (e.g., cold ischemia and reperfusion) and has various cellular functions, including the inhibition of NF-κB activation, macrophage inhibitory factors, apoptosis, and the maintenance of redox homeostasis by acting as an antioxidant, scavenging ROS and preventing oxidative damage by ROS [39,40]. In the kidney, Trx-1 is constitutively expressed in all regions of the nephron, with increased nuclear expression in the proximal tubule under oxidative stress conditions [41]. Heme oxygenase-1 (HO-1) is a rate-limiting enzyme that catalyzes heme degradation to biliverdin, carbon monoxide and free iron, and exhibits antioxidant, anti-inflammatory, and anti-apoptotic effects, as well as other beneficial effects such as the regulation of autophagy and hemodynamics. Unlike Trx-1, which is constitutively expressed, HO-1 expression in the kidney is inducible [42]. Interestingly, while the activation of nuclear factor erythropoietin-2-related factor 2 (Nrf2) is known to induce and upregulate Trx-1 and HO-1 expressions, whose combined action prevents disease progression [43,44], H_2_ had no effect on Nrf2 in the present study, even though Trx-1 and HO-1 expressions were significantly upregulated by H_2_. Given the temporal dynamics of Nrf2 activation and the fact that it peaks and declines very quickly, as it takes less than 15 min from the time of exposure to switch on the nuclear import of Nrf2 [45], it is possible that our 4-h period of cold preservation followed by 4 h of reperfusion missed the window for detecting Nrf2 activation directly, and instead caught its downstream effects (Trx-1 and HO-1 expression). This is followed by the activation of a delayed mechanism that controls switching off Nrf2’s activation of gene expression once the oxidative stress subsides. The low MDA and activated HO-1 and Trx-1 in the present study suggest that by 4 h, H_2_ substantially reduced oxidative stress and probably turned off the Nrf2. It is also possible that a different mechanism such as activator protein-1 (AP-1), a transcription factor whose activation also upregulates Trx-1 and HO-1 expressions and other antioxidant and protective genes under various cellular stressors [46,47], may partly account for the renal graft protection by H_2_ in the present study. However, a relation between AP-1, Trx-1 and HO-1 needs to be well-established under pathological conditions such as cold ischemia and reperfusion in kidney transplantation. As the upstream mechanism driving Trx-1/HO-1 upregulation in our model remains unconfirmed, AP-1 as an alternative regulator should be directly tested in future models.

Another novelty that distinguishes the present study from other studies investigating the potential therapeutic benefits of H_2_ against IRI is our use of a novelly manufactured, shelf-stable H_2_-saturated solution. It is shelf-stable, as its packaging design is impermeable to H_2_ such that no loss will occur during its storage, barring any exterior damage occurring. Being shelf-stable and readily available for use, this preservation solution is rather low-maintenance, and thus well equipped for use under demanding circumstances such as busy hospital settings.

Despite the promising results in the present study suggesting the use of H_2_ in clinical kidney transplantation, our work has limitations. Since we cold-preserved the renal grafts for only 4 h as a proof of principle to ensure that H_2_ therapy worked with a storage time shorter that that used clinically, future studies should focus on a longer preservation time, as well as kidney transplantation in living recipient pigs under immunosuppressive therapy to make the model more clinically applicable. Also, as static cold storage is the gold standard of clinical organ preservation for transplantation, it would be interesting for additional studies to be done using static cold storage and compare the effect of H_2_ under this preservation method to hypothermic pulsatile perfusion as used in the present study. In addition, dose-dependent studies should be considered in future studies to determine the range of optimal doses of H_2_ required to exhibit its therapeutic benefits in the transplantation of kidney and other transplantable solid organs. Furthermore, given that mitochondria are a major contributor to IRI in organ transplantation, we expect mechanistic studies in the future to investigate the effects of H_2_ on mitochondrial systems and other subcellular mechanisms in relation to attenuation of IRI in organ transplantation. Nonetheless, the results of our work add to the growing body of knowledge about the therapeutic impact of H_2_ in transplantation of kidney and other transplantable solid organs and draws this versatile gas closer to clinical organ transplantation.

## 4. Materials and Methods

### 4.1. Ethical Statement

The experimental procedure in this study was approved by the Animal Ethics Committee of the University of Western Ontario, Canada (Protocol ID: 2023-137).

### 4.2. Induction of Warm Ischemia, Hypothermic Pulsatile Perfusion, and Ex Vivo Reperfusion

**Figure 5:**
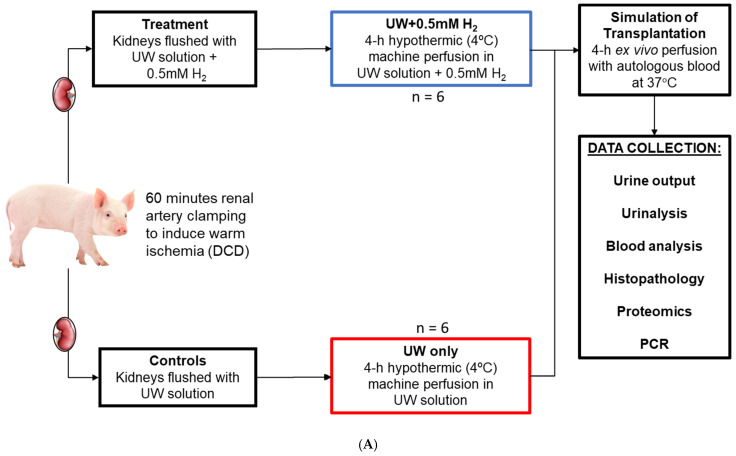

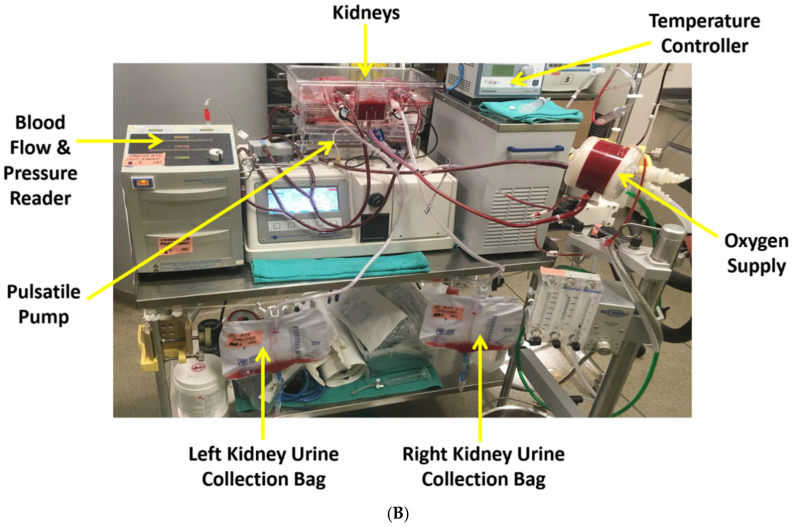
(**A**) Flowchart of experimental design showing UW control group (UW only) and hydrogen-saturated UW group (UW + H_2_). (**B**) Ex vivo pulsatile perfusion setup, showing reperfusion with autologous blood at 37 °C after hypothermic machine perfusion. Control and H_2_-treated renal grafts placed in a perfusion cassette and reperfused with autologous blood. Ureters of both kidneys connected to separate bags for urine collection. Blood temperature regulated with a temperature controller connected to oxygenator.

Male Yorkshire swine (*n* = 9; 60–70 kg) obtained from Lakeview Swine Farms were anesthetized prior to making a midline incision to allow the visualization of the kidneys. To mimic a clinical DCD situation, each renal artery was clamped for 60 min before a nephrectomy was performed. Left and right donor kidneys from 6 pigs were randomly assigned to either the UW-only group (control; *n* = 6) or the hydrogen-saturated University of Wisconsin (UW) solution (UW + H_2_; 0.5 mM H_2_) group (*n* = 6), as illustrated in Figure 5A. Sham-operated pigs (*n* = 3; 6 kidneys) served to reduce biases and evaluate the specific effectiveness of H_2_ treatment. Prior to its addition to UW solution, H_2_ was titrated to determine its concentration using methylene blue as an indicator. The physicochemical properties of H_2_-supplemented UW solutions are listed in Table 1.

Upon nephrectomy, the kidneys were flushed with cold UW solution or cold hydrogen-saturated (0.5 mM) UW solution. The concentration of H_2_ used was determined from a previous study [18]. While flushing was occurring, the pigs were exsanguinated, and 2 L of whole blood were collected into IV bags containing 10,000 units of heparin and temporarily stored at 4 °C. Both kidneys were then connected to a hypothermic (4 °C) pulsatile perfusion pump for 4 h while perfused with UW solution or UW + H_2_ solution. During this time, systemic pressure was maintained at 50 mmHg (±1 mmHg) while flow rate (mL/min) and renal resistance (mmHg*min/mL) were recorded every hour. Two hours prior to the start of reperfusion, previously collected autologous blood was warmed at room temperature and given 1 g Ancef, 5 mg Verapamil, 0.05 g creatinine, and 10,000 units of heparin each to prevent any possible infection and clotting. At the end of the 4-h period of cold preservation by hypothermic machine perfusion, the kidneys were taken off the perfusion pumps and re-flushed with new preservation solutions according to their treatment groups. During this time, the perfusion pumps were flushed with PlasmaLyte solution (Baxter, Deerfield, IL, USA) before introducing the whole blood. After flushing was completed, the kidneys were reconnected to their respective perfusion pumps and perfused with oxygenated whole blood at 37 °C for 4 h to simulate transplantation and analyze renal function (Figure 5B). Blood and urine samples as well as blood flow rates (mL/min) and urine outputs (mL) were collected and recorded hourly. Blood parameters (e.g., lactate, pH, pO_2_, electrolytes) and renal function markers (e.g., creatinine and blood urea nitrogen) were also measured hourly using iSTAT Handheld Blood Analyzer (Abbot Laboratories, Chicago, IL, USA). PlasmaLyte solution was used to compensate for any fluid volume loss during reperfusion. After 4 h of reperfusion, the kidneys were removed from the perfusion pumps, sectioned and stored in 10% neutral-buffered formalin for histopathological analysis or stored in RNAlater solution (Thermofisher Scientific, Waltham, MA, USA) for quantitative reverse transcription polymerase chain reaction (rt-qPCR) analysis.

### 4.3. UW + H_2_ Preservation Solution Preparation

The UW + H_2_ organ preservation solution used in this study was manufactured by Diatomic Inc., Garden Ridge, TX, USA, and the specifics of its novel manufacturing method shall remain undisclosed, as it is proprietary. The solution is made to be shelf-stable and ready-for-use, and its packaging designed to be impermeable to hydrogen gas to ensure no loss during storage.

### 4.4. rt-qPCR Analysis

Total RNA was isolated from renal graft tissue preserved in RNAlater using an RNeasy^®^ Mini Kit (Qiagen, Toronto, ON, Canada) following the provided Quick Start Protocol. The purity of the extracted RNA was determined using a UV spectrophotometer and its consequent absorbance ratio. The RNA was reverse transcribed into cDNA using a OneScript^®^ Plus cDNA Synthesis Kit (ABM, Richmond, BC, Canada) following the manufacturer’s protocol using oligo(dT) primers. Rt-qPCR analysis was conducted with primers designed using PrimerBLAST software version 2.5.0 (NCBI) against interleukin 1 beta (IL-1β), B-cell lymphoma 2 (Bcl-2), heme oxygenase-1 (HO-1), mitogen-activated protein kinase 1 (MAPK1), and peptidylprolyl isomerase A (PPIA; housekeeping primer), as shown in Table 2. Reaction mixtures were made as per Blastaq^®^ Green 2X qPCR Master Mix (ABM, Canada) protocol and analyzed using a QuantStudio3^®^ qPCR System machine (ThermoFisher, Toronto, ON, Canada). All genes of interest were normalized against PPIA, and fold changes were compared to sham-operated porcine kidney tissue and calculated via the −ΔΔCq^2^ method.

### 4.5. Histopathological Examination

Formalin-fixed renal tissues were embedded in paraffin and sliced into 4 μm-thick sections for histopathological examination. Sections were dewaxed and stained with Hematoxylin and Eosin (H&E) and Terminal deoxynucleotidyl transferase dUTP nick end labeling (TUNEL) to assess acute tubular necrosis (ATN) and apoptosis, respectively. The H&E sections were scored by a blinded renal pathologist as per the following previously reported scheme: 1 = <11%, 2 = 11–24%, 3 = 25–45%, 4 = 46–75%, 5 = >75% [7]. Immunohistochemical staining was performed on unstained sections using the following primary antibodies: KIM-1 (tubular injury marker; 1:80; Proteintech, Rosemont, IL, USA), IL-6 (proinflammatory cytokine; 1:50; Proteintech, Rosemont, IL, USA), CD68 (macrophage marker; 1:250; Proteintech, Rosemont, IL, USA), MPO (neutrophil marker; 1:30; Abcam Inc., Toronto, ON, Canada), MDA (oxidative stress marker; 1:30; Abcam Inc., Toronto, ON, Canada), Nrf2 (antioxidant marker; 1:200; Proteintech, Rosemont, IL, USA), HO-1 (stress-responsive marker; 1:50; Proteintech, Rosemont, IL, USA), Trx-1 (cell redox marker; 1:100; Proteintech, Rosemont, IL, USA), NF-κB (inflammation-related transcription factor; 1:150; Insight Biotech Ltd., Welwyn Garden City, UK), and p38 (regulator of inflammatory mediators; 1:50; Cell Signaling Technology, Whitby, ON, Canada). The stained sections were viewed using an Aperio ImageScope (version 12.4; Leica Biosystems, Deer Park, IL, USA) at 10× magnification.

### 4.6. Proteomics Sample Preparation

Sample processing was performed using the single-pot, solid-phase-enhanced sample preparation strategy (SP3) as previously described [48,49]. Briefly, tissue was homogenized in 6 M guanidinium hydrochloride, 0.1 M Tris(hydroxymethyl)aminomethane pH 8 containing 20 mM Tris(2-carboxyethyl)phosphine (TCEP; ThermoFisher Scientific) and 60 mM iodoacetamide (Sigma-Aldrich, Oakville, ON, Canada) at 60 °C, then heated briefly to 95 °C and sonicated by probe-tip using a Sonic Dismembrator Model 100 (FisherScientific, Ottawa, ON, Canada) with a 10 s ON–30 s OFF cycle and amplitude setting of 2, and then incubated for 45 min at 30 °C. Protein extracts were centrifuged at 21,000× *g* for 10 min at room temperature, quantified by a Pierce 660 nm Protein Assay Reagent (ThermoFisher Scientific), then mixed 1:10 with SP3 beads (Cytiva, Burnaby, BC, Canada) and precipitated from an equal volume of 100% ethanol, pooled with peptide digests acidified to 0.5% trifluoroacetic acid (TFA). Peptides were desalted using Oasis HLB 1cc (10 mg; Waters) solid-phase extraction cartridges, eluates were evaporated to dryness by speed vacuum concentration, and the resultant peptides were resuspended in 2% ACN, 0.1% TFA shaking 1400 rpm for 5 min, then an ultrasonic bath for 20 min. Peptide samples were centrifuged at 21,000× *g* for 7 min at room temperature, quantified by microBCA assay (ThermoFisher Scientific), then transferred to Waters Total Recovery autosampler vials for injection.

### 4.7. Liquid Chromatography Mass Spectrometry

Peptide samples were injected onto a Waters Acquity UPLC M-Class system (Waters) coupled to a Q Exactive Plus hybrid quadrupole-Orbitrap mass spectrometer coupled to an Orbitrap Eclipse Tribrid quadrupole-ion trap-Orbitrap mass spectrometer (ThermoFisher Scientific). Samples were trapped (5 μL min^−1^, 5 min) on a Symmetry C18 Trap Column, 5 μm, 180 μm × 20 mm (Waters, Mississauga, ON, Canada) using 99% Mobile Phase A (H2O/0.1% formic acid (FA)) and 1% Mobile Phase B (ACN/0.1% FA), then resolved on a Peptide BEH C18 Column, 130 Å, 1.7 μm, 75 μm × 250 mm (Waters) at 35 °C and a flow rate of 300 nL min^−1^ using a nonlinear gradient (1–7% B, 1 min; 7–23% B, 179 min; 23–35% B, 60 min; 35–98% B, 5 min). The Q Exactive Plus mass spectrometer was controlled by Xcalibur software v4.0 (ThermoFisher Scientific). Data were acquired in data-dependent acquisition mode (DDA) using a FT/FT/HCD (Fourier Transform/higher-energy collision dissociation) Top 12 scheme. Survey scans (MS1) were acquired from *m*/*z* 375 to 1500 at a resolution of 70,000 with AGC (automatic gain control) set to 3 e6 and a maximum injection time of 250 ms. Multiply-charged peptide ions were isolated using a quadrupole isolation window of *m*/*z* 1.2 and were fragmented using HCD with a NCE (normalized collision energy) set to 25%. Fragment ion scans (MS2) were acquired at a resolution of 17,500 with AGC set to 2 × 10^5^ and a maximum injection time of 64 ms. Dynamic exclusion was set to 45 s, and lock mass ion was enabled at *m*/*z* 445.120025. Peptide features were matched by automated retention time alignment with a mass error of 10 ppm and feature intensity ≥ 1 × 10^5^, and filtered for average area ≥ 1 × 10^4^, quality score ≥ 5, and charge state of 1–6, using Top 3 peptides. Proteins were filtered for ≥3 peptides with outlier and modified peptide exclusion, a significance ≥ 20 (ANOVA), and fold-change ≥ 1.2 or ≤0.83 across at least *n* ≥ 3 biological replicates in any one independent experimental grouping.

### 4.8. Statistical Analysis

All statistical analyses were performed using GraphPad Prism 10 (version 10.0.0 for Mac, CA, USA). Data were analyzed using paired T-tests and one-way analysis of variance (ANOVA) to determine statistical differences between treatment groups. Statistical significance was accepted at *p* < 0.05. Values are presented as mean ± standard error of the mean (SEM). A statistical power of 0.80 was computed using the formula: Power = 1 − β, where β is Type-II error probability based on the acceptable risk of missing a real effect.

## 5. Conclusions

In conclusion, the present study provides the first ex vivo report showing that the supplementation of a standard organ preservation solution with H_2_ for hypothermic pulsatile perfusion for 4 h, followed by 4 h of normothermic reperfusion, mitigates renal IRI and improves renal graft viability and function. Additionally, we identified Trx-1/HO-1 as a novel pathway associated with the graft-protecting mechanism of H_2_. Considering the increasing number of DCD kidneys being discarded annually, our work could lay the foundation for more large animal studies and preliminary human clinical trials for the use of H_2_ in the transplantation of kidney and other transplantable solid organs.

## Figures and Tables

**Table 1:** Component of H_2_-supplemented UW solutions.

Solution	Component	Concentration
UW Cold Storage Solution	Potassium lactobionate	100 mmol/L
	KH_2_PO_4_ (monobasic potassium phosphate)	25 mmol/L
	MgSO_4_ (magnesium sulfate)	5 mmol/L
	Raffinose	30 mmol/L
	Adenosine	5 mmol/L
	Glutathione	3 mmol/L
	Allopurinol	1 mmol/L
	Hydroxyethyl starch (HES)	50 g/L
	Sodium	~29 mEq/L
	Potassium	~125 mEq/L
	Osmolarity	~320 mOsm/kg
	pH	~7.4
	Molecular hydrogen (H_2_) at 4 °C	0.5 mM
UW Machine Perfusion Solution	Sodium	~100 mEq/L
	Potassium	~25 mEq/L
	Potassium lactobionate	Less than UW CSS
	Raffinose	Present
	Hydroxyethyl starch (HES)	Less than UW CSS
	Adenosine	Present
	Glutathione	Present
	Allopurinol	Present
	Magnesium sulfate	Present
	Phosphate buffer	Present
	Osmolarity	~300 mOsm/kg
	pH	~7.4
	Viscosity	Less than UW CSS
	Molecular hydrogen at 4 °C	0.5 mM

**Table 2:** Primers used for real-time quantitative PCR and accession numbers of analyzed genes.

Gene Symbol	Accession No.	Primer Sequence (3′ to 5′)	Product Size (bp)
IL-1β	NM_214399.1	F-CAGCACCTCTCAAGCAGAACAA	248
		R-GGCAGCAACCATGTACCAACT	
Bcl-2	XM_021099593.1	F-GGATAACGGAGGCTGGGATG	86
		R-CCTTCAGAGACAGCCAGGAG	
HO-1	NM_001004027.1	F-TACCGCTCCCGAATGAACAC	140
		R-TGGTCCTTAGTGTCCTGGGT	
ERK (MAPK1)	NM_001198922.2	F-CCTTGACTCCTTTGAGCCGT	266
		R-GGTCACTGCTGCCCTAAAGT	
PPIA	NM_214353.1	F-GCGTCTCCTTCGAGCTGTTT	231
		R-CAGGACCCGTATGCTTCAGG	

## Data Availability

The original contributions presented in this study are included in the article.
